# Clinical characteristics and prognosis of heart failure with mid-range ejection fraction: insights from a multi-centre registry study in China

**DOI:** 10.1186/s12872-019-1177-1

**Published:** 2019-09-02

**Authors:** Siqi Lyu, Litian Yu, Huiqiong Tan, Shaoshuai Liu, Xiaoning Liu, Xiao Guo, Jun Zhu

**Affiliations:** State Key Laboratory of Cardiovascular Disease, Emergency and Critical Care Center, Fuwai Hospital, National Center for Cardiovascular Diseases, Chinese Academy of Medical Sciences and Peking Union Medical College, No. 167 Beilishi Road, Xicheng District, Beijing, 100037, People’s Republic of China

**Keywords:** Heart failure, Mid-range ejection fraction, Ischemic heart disease, Prognosis

## Abstract

**Background:**

Heart failure (HF) with mid-range ejection fraction (EF) (HFmrEF) has attracted increasing attention in recent years. However, the understanding of HFmrEF remains limited, especially among Asian patients. Therefore, analysis of a Chinese HF registry was undertaken to explore the clinical characteristics and prognosis of HFmrEF.

**Methods:**

A total of 755 HF patients from a multi-centre registry were classified into three groups based on EF measured by echocardiogram at recruitment: HF with reduced EF (HFrEF) (*n* = 211), HFmrEF (*n* = 201), and HF with preserved EF (HFpEF) (*n* = 343). Clinical data were carefully collected and analyzed at baseline. The primary endpoint was all-cause mortality and cardiovascular mortality while the secondary endpoints included hospitalization due to HF and major adverse cardiac events (MACE) during 1-year follow-up. Cox regression and Logistic regression were performed to identify the association between the three EF strata and 1-year outcomes.

**Results:**

The prevalence of HFmrEF was 26.6% in the observed HF patients. Most of the clinical characteristics of HFmrEF were intermediate between HFrEF and HFpEF. But a significantly higher ratio of prior myocardial infarction (*p* = 0.002), ischemic heart disease etiology (*p* = 0.004), antiplatelet drug use (*p* = 0.009), angioplasty or stent implantation (*p* = 0.003) were observed in patients with HFmrEF patients than those with HFpEF and HFrEF. Multivariate analysis showed that the HFmrEF group presented a better prognosis than HFrEF in all-cause mortality [*p* = 0.022, HR (95%CI): 0.473(0.215–0.887)], cardiovascular mortality [*p* = 0.005, HR (95%CI): 0.270(0.108–0.672)] and MACE [*p* = 0.034, OR (95%CI): 0.450(0.215–0.941)] at 1 year. However, no significant differences in 1-year outcomes were observed between HFmrEF and HFpEF.

**Conclusion:**

HFmrEF is a distinctive subgroup of HF. The strikingly prevalence of ischemic history among patients with HFmrEF might indicate a key to profound understanding of HFmrEF. Patients in HFmrEF group presented better 1-year outcomes than HFrEF group. The long-term prognosis and optimal medications for HFmrEF require further investigations.

**Electronic supplementary material:**

The online version of this article (10.1186/s12872-019-1177-1) contains supplementary material, which is available to authorized users.

## Background

Historically, heart failure (HF) has been classified into two strata: HF with reduced ejection fraction (EF) (HFrEF; EF<40%) and HF with preserved EF (HFpEF; EF ≥ 50%). However, it has been increasingly recognized that HF patients with an intermediate EF (40 to 49%), which represent approximately 10–20% of all HF cases [1–12], may be a clinically distinct group. To better serve this patient population, the 2016 European Society of Cardiology (ECS) guidelines listed HF with mid-range EF (HFmrEF; EF 40–49%) as a separate group, parallel to HFrEF and HFpEF, in order to promote research about its underlying characteristics, pathophysiology and treatment [13].

Numerous clinical studies and trials have been conducted for HFrEF patients. These studies and trials produced a large body of data, providing evidence-based guidance to medications and leading to significant improvement in prognosis [13]. In contrast, despite of a significant number of clinical studies and trials for HFpEF, there is no definitive, effective evidence-based therapy for this HF subpopulation [13]. In past trials, the EF cut-off for HFpEF was variably defined as EF > 40, > 45, > 50% and ≥ 55%. These cut-off values were arbitrary, as there lacked pathophysiologic or clinical evidences to establish the superiority of one cut-off versus another. When the cut-off for HFpEF was set at EF ≥ 50%, patients with intermediate EFs (i.e., EFs between 40 and 49%) were neither HFpEF nor HFrEF. These patients were referred to as HFmrEF and were initially considered as cases of mild systolic HF [13]. While the EF of HFmrEF patients is intermediate between those of HFrEF and HFpEF, increasing evidence suggests that it would be simplistic to view HFmrEF as the transition between HFrEF and HFpEF. Instead, HFmrEF differs from HFrEF and HFpEF not only in clinical characteristics, but also in pathophysiological mechanisms [1–12]. Therefore, HFmrEF should be considered as a distinct HF subpopulation that requires its own evidence-based therapy.

As the distinctiveness of HFmrEF is recognized, research is needed to better characterize and understand this HF subpopulation. Researches on HFmrEF patients in Asian or China are particularly needed, as the majority of previous researches were performed among Western populations. In order to fill the knowledge gap of HFmrEF, especially HFmrEF among Asians, we conducted retrospective analysis on a cluster of HF patients from a multi-centre observational study in China. The demographics, medical history, clinical characteristics, medication status and prognosis of HFmrEF were investigated with respect to those of HFrEF and HFpEF.

## Methods

### Study population

The Chinese HF registry was designed to enroll adult (≥18 years of age) patients with a clinical diagnosis of HF from participating centers between December 2012 and November 2014. A total of 24 sites, evenly distributed in north, south, central, east, west, north-east and north-west regions of China, participated in this registry. These registry sites were selected to represent different levels of medical care (e.g., large hospitals and small hospitals, rural and urban, academic and community). By design, approximately two-thirds of the patients were recruited from outpatient clinics, and the rest one-third from inpatient hospital wards in order to reflect the relative ratio of patients in the real practice setting. The Boston criteria [14] were used to confirm the diagnosis of HF. Patients with a life expectancy less than the duration of the follow-up due to severe non-cardiac diseases, as well as patients who were noncompliant for follow-up visits, were excluded from the study. This study was centrally managed by Fuwai Hospital, Beijing. The study protocol (Protocol Final Version 2.0, 2012-03-20; Ethical Approval Number: 2012–428; Project Number: 2012-ZX013) was approved by the Institutional Review Boards of Fuwai Hospital and conformed to the Declaration of Helsinki. All patients have signed consent for participating this study.

### Baseline

Baseline characteristics of the patients were collected at enrollment, including information on demographics, medical history, symptoms, physical examination, laboratory tests, imaging examination and therapies. Following recommendations of the 2016 ESC guideline [13], participants were stratified into HFrEF (EF < 40%), HFmrEF (EF 40–49%) and HFpEF (EF ≥ 50%) groups according to their left ventricular ejection fraction (LVEF) as measured by quantitative transthoracic echocardiography at baseline.

### Follow-up and outcomes

Trained research personnel conducted a 1-year follow-up via telephone or outpatient service. Data were collected on symptom status and clinical events. The primary endpoint was all-cause mortality and cardiovascular mortality during the 12-month follow-up period. Specifically, deaths and their causes were recorded and ascertained by review of the relevant medical documents while any additional information needed, if not documented, was obtained by contacting one of the patient’s physicians or relatives. Secondary endpoints were defined as hospitalization due to HF and major adverse cardiac events (MACE). Hospitalization due to HF was defined as any new hospitalization with a primary discharge diagnosis for HF. MACE refers to composite endpoint events of cardiovascular mortality, myocardial infarction and stroke, which were identified locally and recorded on the study case report forms.

### Statistical analysis

Continuous variables were presented as medians with 25th to 75th interquartile ranges and compared by Kruskal-Wallis tests. Categorical variables were presented as percentages and compared by Chi-square tests. For multiple comparisons, the Bonferroni correction was used to adjust the significance level. Kaplan-Meier survival curves were constructed for all-cause mortality and cardiovascular mortality while log-rank tests were used to compare the unadjusted survival curves of the three groups. Univariate and multivariate Cox proportional hazard regression were performed to identify the association of the three EF strata with 1-year all-cause mortality and cardiovascular mortality, while logistic regression was performed for hospitalization due to HF and MACE. In the Cox regression and Logistic regression models, hazard ratio (HR) and odds ratio (OR) with 95% confidence intervals (CI) were calculated, respectively. All variables were tested in the univariate analysis. Variables that achieved a *P* value < 0.10 in the univariate models or that were considered clinically relevant with outcomes were entered into the multivariate analysis. To avoid overfitting, backward LR (likelihood ratio) method were performed with retention set at a significance level of 0.10. Subgroup analyses were performed to assess the homogeneity of the association between the three EF strata and 1-year cardiovascular mortality. All statistical tests were 2-sided, and statistical significance was defined as *p* values < 0.05. SPSS 25.0 (IBM Corporation, New York, USA) was used for all statistical analyses.

## Results

**Fig. 1:**
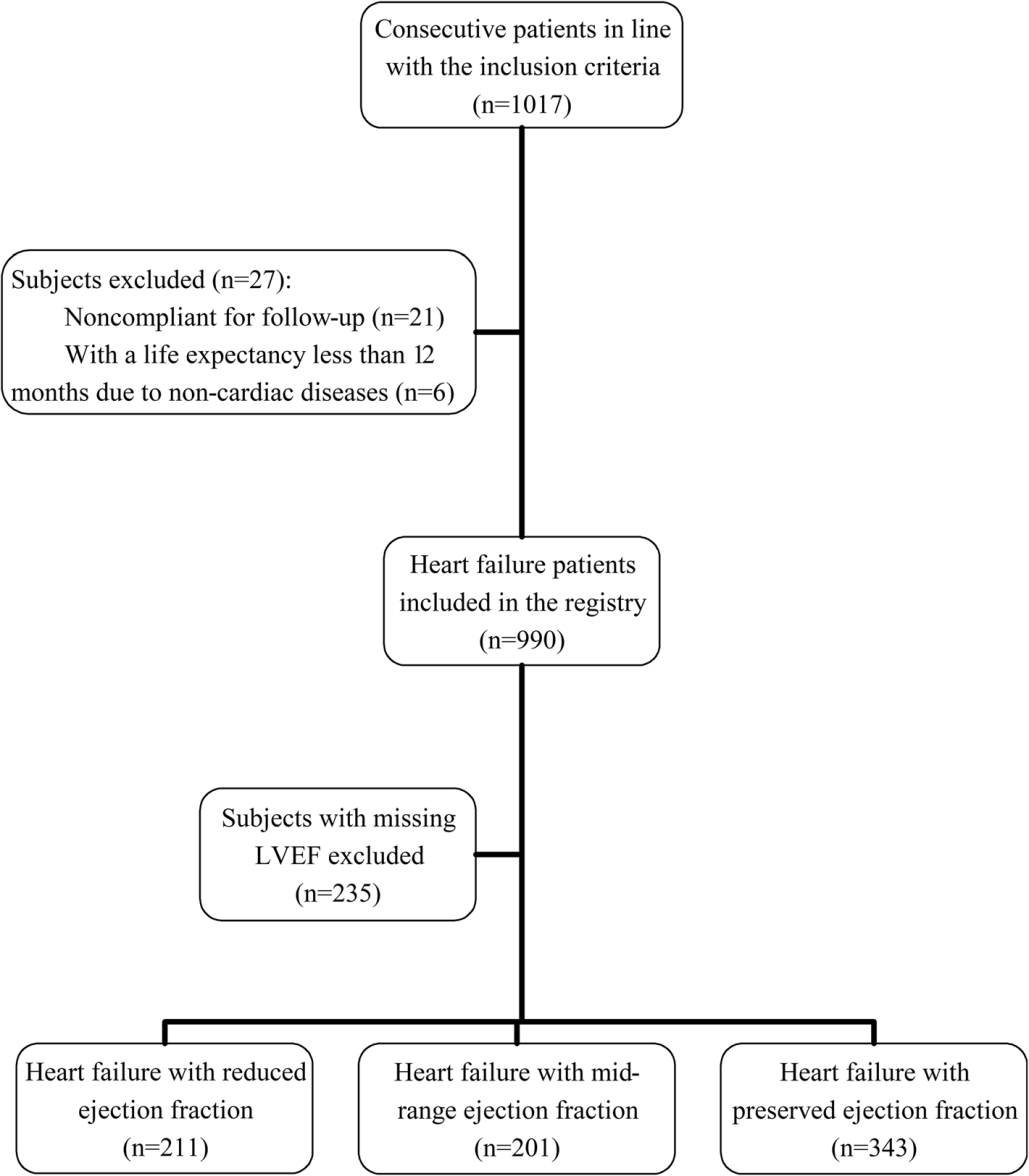
Participant flow chart

Between December 2012 and November 2014, 1017 consecutive patients who accorded with the inclusion criteria were recruited. Among them, 21(2.1%) patients noncompliant for follow-up and 6(0.6%) patients with a life expectancy less than 12 months due to non-cardiac diseases were withdrawn from the study, while baseline assessment and 1-year follow-up of the rest 990 patients were completed. After excluding 235 subjects with missing LVEF, 755 HF patients from 24 hospitals were included in the analysis. Among these patients, 201 (26.6%) were classified as having HFmrEF, 211 (28.0%) had HFrEF, and the rest 343 (45.4%) had HFpEF (Fig. 1). Table 1 shows the baseline characteristics of the three HF groups, including demographics, clinical findings, medical history, etiology, medication status and echocardiogram information. A comparison of the major characteristics of the 755 included patients with the 235 excluded patients is shown in Additional File: Additional file 1: Table S1.

**Table 1:** Baseline Characteristics of HF Population Stratified by LVEF

Characteristic	HFrEF (*n* = 211)	HFmrEF (*n* = 201)	HFpEF (*n* = 343)	*p* value
Demographics, n (%)				
Female	67 (31.8%)^#^	68 (33.8%)^#^	179 (52.2%)*	< 0.001
Age (y)	62 (50–70)^#^	66 (58–75)*	71 (61–77)*	< 0.001
BMI (kg/m^2^)	23.14 (20.96–25.95)	24.43 (21.75–26.65)	24.03 (21.30–26.72)	0.109
Married	192 (91.0%)^#^	178 (88.6%)^#^	268 (78.1%)*	< 0.001
Non-solitary	196 (92.9%)	188 (93.5%)	312 (91.0%)	0.505
Medication insurance	183 (86.7%)	168 (83.6%)	295 (86.0%)	0.63
Educated	144 (68.2%)^#^	125 (62.2%)	198 (57.7%)*	0.046
MoCA	22.81 (19–28)	22.24 (18–27)	21.48 (17–27)	0.058
Clinical findings, n (%)				
Pulse (bpm)	80 (68–90)^#^	74 (64–83.5)*	75 (68–84)*	0.004
SBP (mmHg)	115 (105–130)^#^	130 (110–140)*	130 (118–140)*	< 0.001
NYHA (III-IV)	158 (74.9%)^#^	121 (60.2%)*	176 (51.3%)*	< 0.001
JVP (>6cmH_2_O)	91 (43.1%^a#^	73 (36.3%)^#^	86 (25.1%)*	< 0.001
Medical history, n (%)				
Hypertension	90 (42.7%)^#^	123 (61.2%)*	207 (60.3%)*	< 0.001
Diabetes Mellitus	43 (20.4%)	44 (21.9%)	70 (20.4%)	0.905
Hyperlipidemia	57 (27.0%)	58 (28.9%)	124 (36.2%)	0.049
COPD	11 (5.2%)	18 (9.0%)	37 (10.8%)	0.078
CKD	17 (8.1%)	12 (6.0%)	21 (6.1%)	0.613
Stroke	27 (12.8%)	32 (15.9%)	55 (16.0%)	0.545
Prior MI	35 (16.6%)	55 (27.4%)*^#^	53 (15.5%)	0.002
Tobacco use	92 (43.6%)^#^	93 (46.3%)^#^	99 (28.9%)*	< 0.001
Family history of HF	12 (5.7%)	7 (3.5%)	10 (2.9%)	0.245
Family history of CAD	14 (6.6%)	19 (9.5%)	27 (7.9%)	0.571
Cardiac Surgery	10 (4.7%)	15 (7.5%)	25 (7.3%)	0.431
Devices	20 (9.5%)^#^	8 (4.0%)	14 (4.1%)*	0.014
Angioplasty or stent implantation	23 (10.9%)	45 (22.4%)*^#^	48 (14.0%)	0.003
Cardiac hospitalization	128 (60.7%)^#^	109 (54.2%)^#^	126 (36.7%)*	< 0.001
Etiology,n (%)				
Ischemic heart disease	90 (42.7%)	119 (59.2%)*	174 (50.7%)	0.004
Hypertensive heart disease	10 (4.7%)^#^	25 (12.4%)*^#^	76 (22.2%)*	< 0.001
Dilated cardiomyopathy	79 (37.4%)^#^	25 (12.4%)*^#^	16 (4.7%)*	< 0.001
Congenital heart disease	3 (1.4%)	1 (0.5%)	6 (1.7%)	0.514
Others	29 (13.7%)	31 (15.4%)	71 (20.7%)	0.077
Medication status, n (%)				
Beta- blockers	133 (63.0%)	140 (69.7%)	205 (59.8%)	0.069
On target dose of beta blockers	10 (4.7%)	6 (3.0%)	11 (3.2%)	0.558
On target heart rate	54 (25.6%)	63 (31.3%)	105 (30.6%)	0.353
On target dose or target heart rate	60 (28.4%)	68 (33.8%)	111 (32.4%)	0.465
ACEIs/ARBs	152 (72.0%)	154 (76.6%)^#^	216 (63.0%)	0.002
On target dose of ACEIs/ARBs	31 (14.7%)^#^	35 (17.4%)*	30 (8.7%)*	0.008
MRAs	160 (75.8%)^#^	128 (63.7%)*^#^	159 (46.4%)*	< 0.001
Diuretics	165 (78.2%)^#^	143 (71.1%)^#^	179 (52.2%)*	< 0.001
Nitrate	67 (31.8%)	76 (37.8%)	114 (33.2%)	0.394
Antiplatelet drugs	129 (61.1%)	150 (74.6%)*^#^	220 (64.1%)	0.009
Anticoagulants	19 (9.0%)	25 (12.4%)	43 (12.5%)	0.402
Digoxin	98 (46.4%)^#^	62 (30.8%)*^#^	49 (14.3%)*	< 0.001

*Abbreviations*: *HFrEF* heart failure with reduced ejection fraction, *HFmrEF* heart failure with mid-range ejection fraction, *HFpEF* heart failure with preserved ejection fraction, *BMI* body mass index, *NYHA* New York Heart Function Assessment, *MoCA* Montreal cognitive assessment, *SBP* systolic blood pressure, *DBP* diastolic blood pressure, *JVP* jugular venous pressure, *COPD* chronic obstructive pulmonary disease, *CKD* chronic kidney disease, *MI* myocardial infarction, *HF* heart failure, *CAD* chronic coronary artery disease, *ACEIs* angiotensin-converting enzyme inhibitors, *ARBs* angiotensin receptor blockers, *MRAs* mineralocorticoid receptor antagonists

*p < 0.025 versus HFrEF

^#^*p* < 0.025 versus HFpEF

### Prevalence of HFmrEF

In the Chinese HF registry, patients with HFmrEF represented 26.6% of all HF population. Hospitals in China are designated as primary, secondary or tertiary based on a hospital’s ability to provide medical care, medical education and conduct medical research. The source of patients and severity of diseases were diverse between different hospital tiers. For example, tertiary hospitals, located in urban centers and staffed by large numbers of specialists, treat a higher concentration of urban patients as well as patients with more serious conditions. When broken down by the tier of hospitals, the prevalence of HFmrEF in the Chinese HF registry was 25.5, 28.5 and 26.1% in primary, secondary and tertiary hospitals, respectively. There was no statistically significant difference between hospital tiers (*p* = 0.745). The rates of patients with HFmrEF were comparable in rural and urban areas (23% vs 27.1%, *p* = 0.415). In the meantime, the differences between northern (29.8%), southern (28.2%), central (31.1%), eastern (24.2%), western (26.2%), north-eastern (23.5%) and north-western (19.5%) China were also not significant (*p* = 0.554). However, the patients recruited from outpatient clinics displayed a slightly higher proportion of HFmrEF than those enrolled from inpatient units (29.2% vs 22.6%, *p* = 0.047). (Shown in Additional file 4: Figure S1).

### Demographic and clinical characteristics of HFmrEF

Comparison of the demographics and medical history of HFrEF, HFpEF and HFmrEF showed that most of the clinical (Table 1) and echocardiographic (Table 2) characteristics of HFmrEF were intermediate between HFrEF and HFpEF. Thereinto, in respective of age, New York Heart Function Assessment (NYHA), rate of hypertension, atrial or ventricular enlargement, left ventricular diastolic dysfunction and valve abnormity, the characteristics of HFmrEF were significantly different from those of HFrEF (all *p* < 0.05) but more similar to those of HFpEF. However, compared with HFpEF, patients with HFmrEF had a higher tendency to be male, married and had a higher rate of cardiovascular hospitalization, tobacco use, abnormal jugular venous pressure (JVP) and medications (including angiotensin-converting enzyme inhibitors or angiotensin II-receptor blockers (ACEIs/ARBs), mineralocorticoid receptor antagonists (MRAs) and diuretics) (all *p* < 0.05), which resembled HFrEF.

**Table 2:** Baseline Echocardiogram Information of HF Population Stratified by LVEF

Parameters	HFrEF(*n* = 211)	HFmrEF(*n* = 201)	HFpEF(*n* = 343)	*p* value
2-dimensional parameters				
LV enlargement (LVEDD>55 mm(Male), 50 mm(Female)), n(%)	188 (89.1%)^#^	136 (67.7%)*^#^	96 (28.0%)*	< 0.001
LV end-diastolic diameter (mm)	61 (48–68)	56 (46–61)	47 (41–57)	
LV end-diastolic volume (ml)	176 (123–189)	146 (117–161)	99 (90–123)	
LA enlargement (LAD>39 mm), n(%)	176 (83.4%)^#^	125 (62.2%)*^#^	177 (51.6%)*	< 0.001
LA diameters	42 (36–47)	41 (34–46)	39 (32–45)	
RV enlargement (RVEDD>25 mm), n(%)	65 (30.8%)^#^	22 (10.9%)*	39 (11.4%)*	< 0.001
RV systolic dysfunction (TAPSE< 16 mm), n(%)	27 (12.8%)^#^	9 (4.5%)*	20 (5.8%)*	0.002
RA enlargement (RAD>40 mm), n(%)	83 (39.3%)^#^	48 (23.9%)*	82 (23.9%)*	< 0.001
Doppler parameters				
LV diastolic dysfunction (E/A ratio < 0.8), n(%)	19 (9.0%)^#^	28 (13.9%)^#^	78 (22.7%)*	< 0.001
Pulmonary hypertension (PASP>40 mmHg), n(%)	60 (28.4%)^#^	38 (18.9%)	66 (19.2%)*	0.021
Valve abnormity, n(%)	112 (53.1%)^#^	76 (37.8%)*	123 (36.9%)*	< 0.001

*Abbreviations*: *LV* left ventricle, *LVEDD* left ventricular end diastolic diameter, *LA* left atrium, *LAD* left atrial diameter, *RV* right ventricle, *RVEDD* right ventricular end diastolic diameter, *TAPSE* tricuspid annular plane systolic excursion, *RA* right atrium, *RAD* right atrial diameter, *E/A* mitral early (E) wave velocity/mitral late (A) wave velocity, *PASP* pulmonary artery systolic pressure

*p < 0.025 versus HFrEF

^#^*p* < 0.025 versus HFpEF

**Fig. 2:**
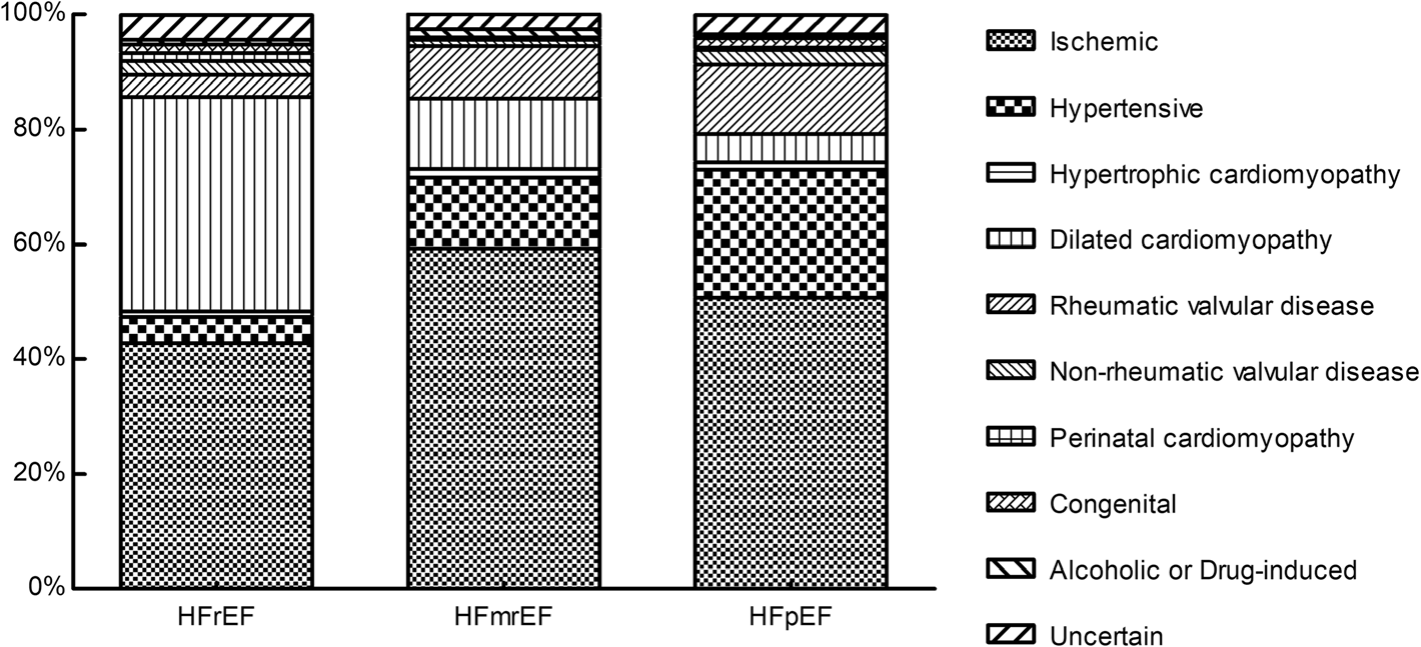
The etiology of HF

Examination of the distribution of HF etiology showed that patients with HFpEF exhibited significantly higher rate of hypertensive heart disease (*p* < 0.001), while patients with HFrEF had a rather higher rate of dilated cardiomyopathy (*p* < 0.001) (Table 1, Fig. 2). Interestingly, the prevalence of ischemic heart disease was significantly higher in patients with HFmrEF than in patients with HFrEF or HFpEF (*p* < 0.001). The rates of prior myocardial infarction (MI) (27.4% vs 15.5 and 16.6%, *p* = 0.002), antiplatelet drug use (74.6% vs 64.1 and 61.1%, *p* = 0.009), angioplasty or stent implantation (22.4% vs 14.0 and 10.9%, *p* = 0.003) in the HFmrEF group were all significantly higher than in the other two groups. These characteristics might indicate the profound essence of HFmrEF.

For patients with HFrEF, beta-blockers, ACEIs/ARBs and MRAs were used in 63, 72 and 75.8%, respectively. Although evidences concerning the management of HFmrEF and HFpEF were lacking, a large proportion of patients with HFmrEF and HFpEF were in the treatment of beta-blockers, ACEIs/ARBs and MRAs. However, in patients who have used beta-blockers, only about one-half have achieved target dose or target heart rate, among whom very few have received optimal dose recommended by guidelines [13, 15]. The percentages of patients receiving recommended dose of ACEIs/ARBs [13, 15] were also very limited. (Table 1).

### 1-year outcomes

**Fig. 3:**
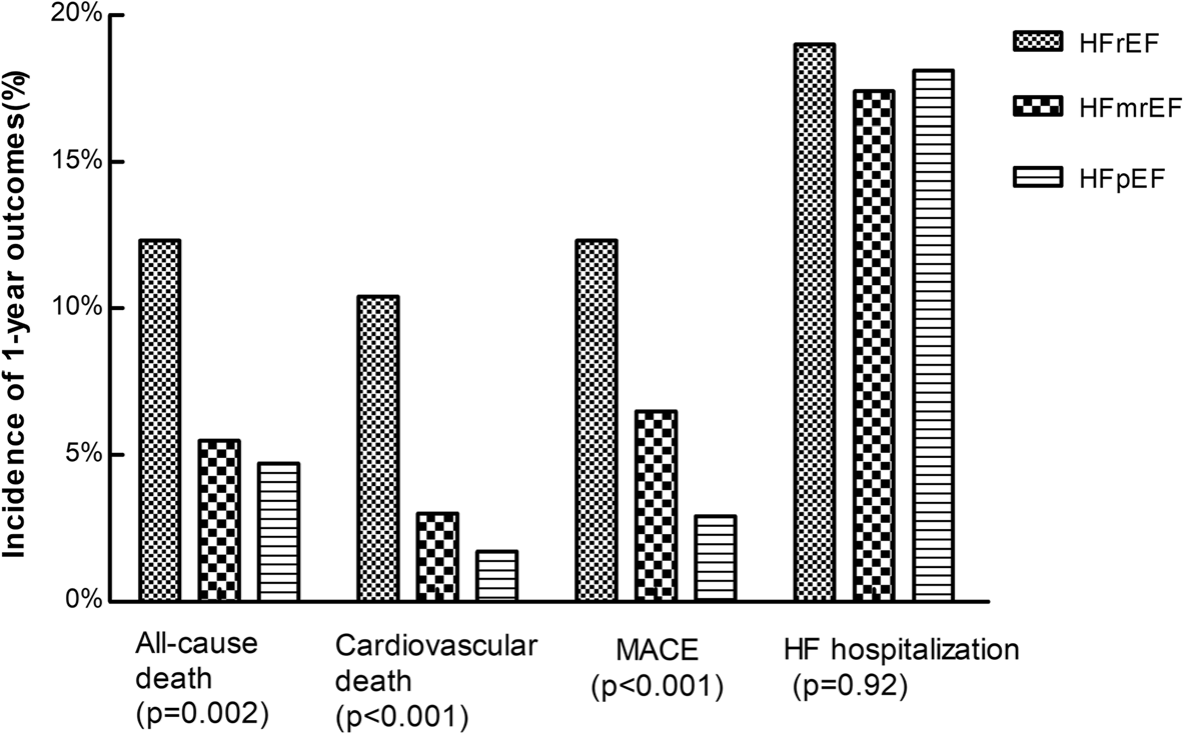
1-year outcomes of HFrEF, HFmrEF and HFpEF

Table 3 and Fig. 3 showed the 1-year outcomes of the three groups. The 1-year all-cause mortality was significantly higher in patients with HFrEF than in those with HFmrEF and those with HFpEF (12.3% vs. 5.5 and 4.7%, *p* = 0.002), so was the 1-year cardiovascular mortality (10.4% vs. 3.0 and 1.7%, *p* < 0.001). The rate of integrated MACE incidence during the 1-year follow-up was 12.3% in HFrEF patients, 6.5% in HFmrEF patients and 2.9% in HFpEF patients (*p* < 0.001). However, there was no significant difference in the three groups’ incidence of hospitalization due to HF (*p* = 0.92).

**Table 3:** 1-Year Outcomes in HF Population Stratified by LVEF

	HFrEF (*n* = 211)	HFmrEF (*n* = 201)	HFpEF (*n* = 343)	*p* value	Univariate analysis				
					HFmrEF vs HFrEF		HFmrEF vs HFpEF		
					HR/OR (95% CI)	*p* value	HR/OR (95% CI)	*p* value	
All-cause Mortality	26 (12.3%)	11 (5.5%)	16 (4.7%)	0.002	**0.461 (0.227–0.935)**	**0.032**	1.233 (0.572–2.660)	0.593	
Cardiovascular Mortality	22 (10.4%)	6 (3.0%)	6 (1.7%)	< 0.001	**0.298 (0.120–0.737)**	**0.009**	1.782 (0.574–5.533)	0.317	
MACE	26 (12.3%)	13 (6.5%)	10 (2.9%)	< 0.001	**0.492 (0.245–0.987)**	**0.046**	2.303 (0.991–5.353)	0.053	
Hospitalization due to HF	40 (19.0%)	35 (17.4%)	62 (18.1%)	0.92	0.901 (0.546–1.488)	0.685	0.956 (0.605–1.509)	0.845	

*Abbreviations*: *HFrEF* heart failure with reduced ejection fraction, *HFmrEF* heart failure with mid-range ejection fraction, *HFpEF* heart failure with preserved ejection fraction, *MACE* major adverse cardiac events

Statistically significant variables were highlighted in bold

**Fig. 4:**
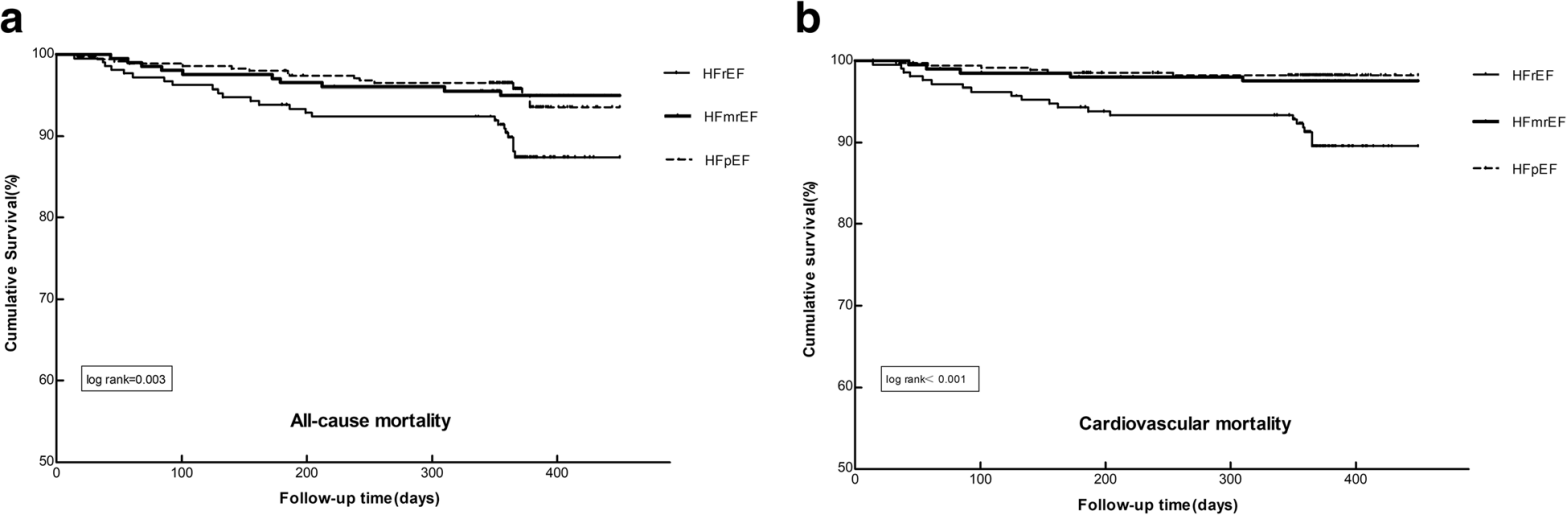
The Kaplan-Meier survival hazard curves of Three HF Groups

Univariate Cox analysis (Table 3) showed that in comparison with HFrEF, HFmrEF was associated with decreased risk of all-cause mortality [*p* = 0.032, HR (95%CI):0.461(0.227–0.935)], cardiovascular mortality [*p* = 0.009, HR (95%CI):0.298(0.120–0.737)] and MACE [*p* = 0.046, OR (95%CI):0.492(0.245–0.987)], whereas the difference between HFmrEF and HFpEF was insignificant. All variables that were thought to have an impact on HF prognosis were tested in the univariate Cox analysis and the results were exhibited in Additional file 2: Table S2. Variables included in multivariate Cox regression were carefully selected in consideration of the number of available events. Only variables with a *p* value <0.10 in univariate analysis (HF categories, non-solitary, MoCA, pulse, SBP, NYHA, abnormal JVP, diabetes mellitus, tobacco use, CKD, stroke, family history of HF, family history of coronary artery disease (CAD), cardiac hospitalization, Ischemic heart disease, ACEIs/ARBs and diuretics) or variables considered clinically relevant with outcomes (age, gender) were included in the backward stepwise multivariate analysis. The final multivariate Cox regression models (Table 4) revealed the same trend after adjusting for common factors related with outcomes: HFmrEF had a better prognosis than HFrEF in all-cause mortality [*p* = 0.022, HR (95%CI): 0.473(0.215–0.887)], cardiovascular mortality [*p* = 0.005, HR (95%CI): 0.270(0.108–0.672)] and MACE [*p* = 0.034, OR (95%CI): 0.450(0.215–0.941)], but was comparable with HFpEF. 1-year Kaplan-Meier survival curves of the three EF strata present significant differences in all-cause mortality (*p* = 0.003) and cardiovascular mortality (*p* < 0.001) (Fig. 4).

**Table 4:** Independent predictors of 1-year events by multivariate Cox analysis

	HFmrEF vs HFrEF			HFmrEF vs HFpEF		
	*p* value	HR/OR (95% CI)		*p* value	HR/OR (95% CI)	
All-cause mortality^a^	**0.022**	**0.437 (0.215–0.887)**		0.488	1.320 (0.602–2.894)	
Cardiovascular mortality^b^	**0.005**	**0.270 (0.108–0.672)**		0.455	1.554 (0.488–4.950)	
MACE^c^	**0.034**	**0.450 (0.215–0.941)**		0.093	2.138 (0.882–5.183)	
Hospitalization due to HF^d^	0.952	1.017 (0.588–1.758)		0.199	0.721 (0.438–1.187)	

*Abbreviations*: *HFrEF* heart failure with reduced ejection fraction, *HFmrEF* heart failure with mid-range ejection fraction, *HFpEF* heart failure with preserved ejection fraction, *MACE* major adverse cardiac events

^a^Final multivariate model adjusted for sex, non-solitary, Montreal cognitive assessment, tobacco use, angiotensin-converting enzyme inhibitors or angiotensin II-receptor blockers

^b^Final multivariate model adjusted for sex, non-solitary, abnormal jugular venous pressure, diabetes mellitus, tobacco use

^c^Final multivariate model adjusted for sex, non-solitary, New York Heart Function Assessment, diabetes mellitus, chronic kidney disease, stroke, tobacco use

^d^Final multivariate model adjusted for New York Heart Function Assessment, abnormal jugular venous pressure, pulse, systolic blood pressure, chronic kidney disease, stroke, family history of HF, cardiac hospitalization

Statistically significant variables were highlighted in bold

Results of subgroup analyses were consistent with those for overall patients. Patients with HFmrEF demonstrated a lower 1-year cardiovascular mortality than those with HFrEF in all subgroups (Table 5). There were no significant interactions between HF categories and age (*p* = 0.538), genders (*p* = 0.352).

**Table 5:** Subgroup analyses for 1-year cardiovascular mortality stratified by EF

	HFmrEF vs HFrEF		HFmrEF vs HFpEF		*p* value for interaction
	HR (95% CI)		HR (95% CI)		
Age<65	**0.114 (0.015–0.881)**		1.164 (0.069–19.545)		0.538
Age ≥ 65	**0.320 (0.103–0.999)**		1.962 (0.549–7.012)		
Male	**0.328 (0.113–0.951)**		4.952 (0.566–43.361)		0.352
Female	**0.103 (0.102–0.846)**		0.456 (0.052–3.971)		

*Abbreviations*: *HFrEF* heart failure with reduced ejection fraction, *HFmrEF* heart failure with mid-range ejection fraction, *HFpEF* heart failure with preserved ejection fraction

Adjusted for age, sex, non-solitary, abnormal jugular venous pressure, diabetes mellitus, tobacco use

Statistically significant variables were highlighted in bold

## Discussion

This retrospective, observational, multi-centre study provided a wealth of information about Chinese patients with HFmrEF. While most of the clinical characteristics of patients with HFmrEF were intermediate between those of HFrEF and HFpEF, HFmrEF displayed a significantly higher rate of ischemia. Both HFmrEF and HFpEF patients had better prognosis in 1-year follow-up than HFrEF.

At present, LVEF is still the most frequently used parameter in the classification of HF [16]. Clinical studies historically stratified HF into HFrEF and HFpEF by LVEF. There was a long history of researches on HFrEF. Consequently, the characteristics, prognosis and therapeutic evidences for HFrEF have been explored extensively, and the research results have been used to improve clinical practice. With the development of further research, HFpEF is gradually recognized as a crucial component of the HF population. A large body of studies, many of which conducted in the recent two decades, reveal that HFpEF differ from HFrEF not only in clinical characteristics but also in pathophysiology mechanisms. Therefore, HFpEF and HFrEF have different prognosis and require distinct medications. The EF cutoff that used to separate HFrEF and HFpEF varied from study to study, creating a “grey area” - HF with EF 40–49% - that was grouped with HFrEF in some studies but with HFpEF in others. In recent years, accumulating evidence suggests that HF with this mid-range EF, now known as HFmrEF, represents a distinct clinical subgroup. However, in-depth studies focused on HFmrEF are limited in quantity and scale compared to studies of HFrEF and HFpEF. Detailed analysis of this Chinese HF Registry did us a favour in further understanding of HFmrEF.

In this multi-centre registry of Chinese heart failure patients, HFmrEF made up about 26.6% of the patients in the registry, which was a little higher than previous reported percentages of HFmrEF (10–20%) [1–11]. This discrepancy might be due to difference in the subject source. Approximately 2/3 of the participants in the Chinese HF Registry were enrolled from outpatient clinics by design to accord with the local practice setting, whereas most of the previous HF trials were confined to hospital inpatient settings, which might have a higher percentage of HFrEF. Comparison of patients recruited from outpatient clinics and inpatient units demonstrated that the former subgroup contained a higher percent of patients with HFmrEF than the latter subgroup, which could help verify this opinion. In the analysis of the ESC-HF-LT Registry, which enrolled 4/7 patients from outpatient clinics, 24.2% of HF patients were classified as having HFmrEF, similarly higher than other publications [8]. Despite the difference in percentage, all studies show that HFmrEF is a substantial proportion of HF family.

Previous studies and clinical trials have preliminarily demonstrated that the clinical characteristics of HFmrEF were intermediate between those of HFrEF and HFpEF, and closer to those of HFpEF in several aspects [1–12]. The GWTG-HF program was one of the largest registry that provided information about HFmrEF. Through analysis of 99,825 HF patients admitted from 305 hospitals, it revealed that patients with HFmrEF possessed medium characteristics between HFrEF and HFpEF in terms of age, gender, comorbidities (e.g., hypertension, anemia, atrial fibrillation/flutter, stroke, depression) and medications. However, the HFmrEF group displayed a higher rate of comorbid ischemic heart disease [4, 9]. Other trials involving HFmrEF (e.g. SwedeHF, ESC-HF-LT) made similar observations [1–12]. The results of our analysis of the Chinese HF Registry were consistent with these previous studies. Most of the baseline characteristics of HFmrEF in the Chinese HF Registry were intermediate between HFrEF and HFmrEF, but might be closer to either one in some respects. The exception was ischemic history (i.e. etiology, history of prior MI, antiplatelet drug use and angioplasty or stent implantation), which showed significantly higher prevalence in the HFmrEF group than in the other two groups. The ratio of ischemic history in patients with HFrEF was lower than most Western studies [1–12], but close to another Chinese observation [2, 17], which might be relevant with the high rate of dilated cardiomyopathy in Chinese HFrEF group. The remarkable feature of HFmrEF might provide clues to its underlying pathophysiology. Previous HF researches have shown that CAD was associated with greater deterioration of ventricular function and transition from HFpEF to HFrEF [18–20]. It has been hypothesized that HFmrEF patients were patients with ischemic heart disease caught in the transition between HFrEF and HFpEF. The LVEF of HFmrEF patients might recover partially with successful anti-ischemic therapy, or go worse because of new-onset ischemic events [19, 20]. To test this hypothesis, longitudinal studies designed to investigate the impact of evidence-based anti-ischemic medications on improving LVEF in patients with HFmrEF are required. Overall, burden of comorbidities in our study was lighter than previous observations [1–11] but parallel to the ESC-HF-LT Registry [12], which could be ascribed to the similar population composition of our study and the ESC-HF-LT Registry [12].

As to echocardiographic characteristics, researches that focused on HFmrEF were limited. He KL, et al. examined the echocardiographic and hemodynamic features of 357 HF patients in a Chinese cohort. It revealed that HF patients with mildly decreased EF (40–55%) demonstrated intermediate characteristics in LV contractility, LV diastolic stiffness and LA dimension between HF patients with normal EF (> 55%) and those with moderate to severely decreased EF (< 40%) [2]. In our registry, rates of left/right ventricular/atrial enlargement, LV diastolic dysfunction, pulmonary hypertension and valve abnormity in patients with HFmrEF were midway between those with HFrEF and those with HFpEF, which was in accordance with previous observations.

As to evidence-based medications of HFrEF, the rates of beta-blockers, ACEIs/ARBs and MRAs usage were lower than those reported in western countries [8, 11], let alone the percent of patients on target dose. On the contrary, there were relatively high rates of digoxin usage in this study, although it has no longer been recommended as a first-line treatment in guidelines [13, 15]. In fact, numerous researches have indicated that digoxin could not improve the outcomes of HF patients [21], or could even worsen their prognosis [22]. The reasons for defects in the management of Chinese HF patients were complicated, which might be related to the limited health care resources, insufficient physician education, relatively low rates of medication insurance, etc. [23, 24]. Efforts should be made to optimizing medications of HF in the future. The management of HFmrEF has not been specifically recommended in existent guidelines. Since patients with HFmrEF were mostly included in trials of HFpEF, it was taken for granted that medications for HFmrEF should abide by the treatment principles of HFpEF rather than those of HFrEF. However, in the “real-world” studies, use of beta-blockers, ACEIs, ARBs and MRAs was quite high in patients with HFmrEF [1–12]. In the Chinese HF Registry, patients with HFmrEF were more likely to be treated with diuretics and digoxin than patients with HFpEF. The rates of prescription of beta-blockers, ACEIs/ARBs and MRAs in patients with HFmrEF were 69.7, 76.6 and 63.7% respectively, all significantly higher than those with HFpEF. In fact, to date, several respective analyses have showed that patients with HFmrEF might benefit from drugs such as beta-blockers, ACEIs, ARBs and MRAs. Cleland et al. performed a meta-analysis of 11 randomized trials and indicated that beta-blockers improved prognosis for patients with HFrEF and HFmrEF in sinus rhythm [25]. Post hoc analysis of the Swedish Heart Failure registry suggested that ACEIs and ARBs were associated with reduced all-cause mortality in HFmrEF (HR 0.85, 95%CI 0.76–0.95) [26]. Moreoever, in the TOPCAT trial, patients with an EF between 45 and 55% benefited from spironolactone treatment [27]. These studies reinforce the opinion that ischemia plays an important role in HFmrEF. Nevertheless, although these studies suggested potential benefits of pharmaceutical interventions in HFmrEF, it should be noted that they were all observational studies. Randomized controlled trials are needed to confirm the benefits of these drugs and determine targeted medical therapies for the management of HFmrEF.

With regard to prognosis, the outcomes of HFmrEF such as mortality and MACE in former studies were shown to be mostly intermediate between HFrEF and HFpEF and closer to those of HFpEF [1–12, 28]. Consistent with previous researches, the 1-year outcomes of patients with HFmrEF in the present study were intermediate between HFpEF and HFrEF. Univariate and multivariate Cox analysis revealed that HFmrEF was associated with a much lower incidence of all-cause mortality, cardiovascular mortality and MACE than HFrEF, while the difference between HFmrEF and HFpEF was not significant. In general, the prognosis of HFmrEF was significantly better than HFrEF and close to HFpEF. Moreover, subgroup analyses revealed that the lower 1-year cardiovascular mortality of HFmrEF in comparison with HFrEF was constant in all subgroup patients (with age<65 or age ≥ 65, male or female). No significant interactions existed between HF categories and age or gender. Previous reports on the rate of HF hospitalization in patients with HFrEF, HFmrEF and HFpEF were controversial [1, 3, 8, 9, 11, 12, 29]. In our registry, the difference in the rate of HF hospitalization during 1-year follow-up were not significant between the three HF groups. Multivariate Cox analysis revealed that NYHA, abnormal JVP, pulse, SBP, CKD and prior cardiac hospitalization were significantly associated with the rate of HF hospitalization (Shown in Additional file 3: Table S3). It’s reasonable that clinical cardiac function, rather than ejection fraction, could predict HF hospitalization during follow-up. Actually, in clinical practice, both patients and doctors pay more attention to clinical symptom rather than ejection fraction in the process of deciding whether to be hospitalized [13, 15]. On the other hand, the severity and frequency of clinical cardiac function deterioration or symptom exacerbation in HFmrEF and HFpEF were not better than HFrEF according to previous researches. In addition, patients with HFpEF and HFmrEF generally have more comorbidities than patients with HFrEF, which might participate in the deterioration of HF symptoms and lead to hospitalization [1, 3, 8, 9, 11–13, 15, 29]. All of these might account for the comparable rate of hospitalization due to HF in our study.

As one of the few investigations focused on Chinese patients with HFmrEF, this study added a significant amount of information about the characteristics and prognosis of HFmrEF. On the other hand, the study had several limitations that need to be noted. Firstly, the study was observational and post hoc, thus causal relationship could not be inferred, and observed variables might have confounded the results. Secondly, only patients who had echocardiograms data were included in the analysis, and a large amount of patients with missing EF values were excluded from the present investigation. Although baseline comparisons between the eligible and the excluded patients presented no remarkable difference, the accuracy of results might be affected nonetheless. Due to limitation in information collection, it’s a pity that some data (such as the exact values of right ventricular end diastolic diameter, right atrium diameter and pulmonary artery systolic pressure) were not recorded, but the percentages of patients with abnormal parameters were listed alternatively. Thirdly, previous researches have revealed a close relationship between human immunodeficiency virus (HIV) infection and heart failure [30]. However, in our registry, the prevalence of HIV infection was 0%. The absence of HIV infection might be relative with Chinese health policy which demanded HIV infection patients to be treated in infectious disease specialist hospitals. This kind of hospitals were not included in our registry, which might affect our comprehensive understanding of the relationship between HIV infection and HF subtypes. Fourthly, although the multivariate analysis had been adjusted for multiple potential confounders, the list of precipitating factors for adverse outcomes could hardly be exhaustive. This might also influence the results. In addition, the follow-up time was relatively short and the incidence of the primary and secondary endpoints were relatively low, which might limit the statistical power. Therefore, more studies with larger sample size and longer follow-up period are needed to confirm our results. Finally, our study only analyzed the LVEF measured at admission and did not have serial EF data during the follow-up, thus we were not able to explore how changes of EF might have affected the outcomes. It has been observed in previous studies that changes of EF were quite common during follow-up and had a significant influence on the prognosis of the patients [12, 28, 29]. Therefore, the variation of EF might be more important than EF itself. What’s more, in order to explore the transition of the three EF strata and evaluate the efficacy of anti-ischemic therapy on EF, it would be essential to measure LVEF at different time points and detect its variation trend. Further effort should be made to propel research in these directions, which might contribute to reveal the intrinsic qualities of HFmrEF.

## Conclusion

Our analysis of the Chinese HF Registry demonstrated that HFmrEF is a subgroup of HF with distinctive characteristics compared to HFrEF and HFpEF, especially in a strikingly prevalence of ischemic history. The 1-year outcomes of Chinese HFmrEF patients were close to that of HFpEF and superior to that of HFrEF. Age and gender did not influence the relationship between HF categories and cardiovascular mortality. Whether HFmrEF is the transition stage of ischemic heart disease between HFpEF and HFrEF needs to be confirmed through more research and longer follow-ups.

## Additional files

Additional file 1: **Table S1.** Baseline characteristics of HF patients included and those excluded. *(Display of the baseline characteristics of HF patients included and excluded) (DOC 55 kb)*

Additional file 2: **Table S2.** Predictors of 1-year events by univariate analysis. *(Display of the predictors of 1-year events by univariate analysis) (DOC 67 kb)*

Additional file 3: **Table S3.** Independent predictors of 1-year events in final multivariate models by backward LR method. *(Display of the independent predictors of 1-year events in final multivariate models by backward LR method) (DOC 44 kb)*

Additional file 4: **Figure S1.** Proportion of three HF groups. *(Description about the proportion of three HF groups in our registry) (TIF 2245 kb)*

## Data Availability

The datasets used and analyzed during the current study are available from the corresponding author on reasonable request.

## Abbreviations

ACEIs: Angiotensin-converting enzyme inhibitors
ARBs: Angiotensin receptor blockers
BMI: Body mass index
CAD: Chronic coronary artery disease
CI: Confidence intervals
CKD: Chronic kidney disease
COPD: Chronic obstructive pulmonary disease
DBP: Diastolic blood pressure
E/A: Mitral early (E) wave velocity/mitral late (A) wave velocity
EF: Ejection fraction
HF: Heart failure
HFmrEF: Heart failure with mid-range ejection fraction
HFpEF: Heart failure with preserved ejection fraction
HFrEF: Heart failure with reduced ejection fraction
HIV: Human immunodeficiency virus
HR: Hazard ratio
JVP: Jugular venous pressure
LA: Left atrium
LAD: Left atrial diameter
LV: Left ventricle
LVEDD: Left ventricular end diastolic diameter
MACE: Major adverse cardiac events
MI: Myocardial infarction
MoCA: Montreal cognitive assessment
MRAs: Mineralocorticoid receptor antagonists
NYHA: New York heart function assessment
OR: Odds ratio
PASP: Pulmonary artery systolic pressure
RA: Right atrium
RAD: Right atrial diameter
RV: Right ventricle
RVEDD: Right ventricular end diastolic diameter
SBP: Systolic blood pressure
TAPSE: Tricuspid annular plane systolic excursion
