# Supplementary material for: Clinical characteristics and prognosis of heart failure with mid-range ejection fraction: insights from a multi-centre registry study in China
Source: BMC Cardiovasc Disord. 2019 Sep 2;19:209. doi: 10.1186/s12872-019-1177-1 (PMC6720401; doi:10.1186/s12872-019-1177-1)
Supplement: Supplementary file 1 — Table S1. Baseline characteristics of HF patients included and those excluded. (Display of the baseline characteristics of HF patients included and excluded) (DOC 55 kb) [file 12872_2019_1177_MOESM1_ESM.doc]

| **Supplementary Table 1. Baseline Characteristics of HF Patients Included and Those Excluded** | | | |
| --- | --- | --- | --- |
| **Characteristic** | **Patients included (n=755)** | **Patients excluded (n-235)** | **p value** |
|
| **Demographics, n (%)** | | | |
| Female | 314(41.6%) | 109(46.4%) | 0.195 |
| Age (y) | 66.9(57.7-75.1) | 71.1(63.2-77.9) | <0.001 |
| BMI (kg/m^2^) | 23.8(21.3-26.5) | 24.0(21.8-26.7) | 0.442 |
| Married | 638(84.5%) | 193(82.1%) | 0.386 |
| Non-solitary | 696(92.2%) | 211(89.8%) | 0.247 |
| Medication insurance | 646(85.6%) | 184(78.3%) | 0.008 |
| Educated | 467(61.9%) | 100(42.6%) | <0.001 |
| MoCA | 24(18-28) | 23(15-27) | 0.093 |
| **Clinical findings, n (%)** | | | |
| Pulse (bpm) | 76(67-85) | 75(66-85) | 0.428 |
| SBP (mmHg) | 124(110-140) | 130(120-140) | <0.001 |
| NYHA (III-IV) | 455(60.3%) | 117(49.8%) | 0.005 |
| JVP (>6cmH_2_O) | 250(33.1%) | 60(25.5%) | 0.029 |
| **Medical history, n (%)** | | | |
| Hypertension | 420(55.6%) | 146(62.1%) | 0.079 |
| Diabetes Mellitus | 157(20.8%) | 58(24.7%) | 0.207 |
| Hyperlipidemia | 239(31.7%) | 74(31.5%) | 0.962 |
| COPD | 66(8.7%) | 30(12.8%) | 0.069 |
| CKD | 50(6.6%) | 16(6.8%) | 0.92 |
| Stroke | 114(15.1%) | 48(20.4%) | 0.054 |
| Prior MI | 143(18.9%) | 39(16.6%) | 0.418 |
| Tobacco use | 284(37.6%) | 82(34.9%) | 0.45 |
| Family history of HF | 29(3.8%) | 11(4.7%) | 0.568 |
| Family history of CAD | 60(7.9%) | 24(10.2%) | 0.276 |
| Cardiac Surgery | 50(6.6%) | 8(3.4%) | 0.067 |
| Devices | 42(5.6%) | 8(3.4%) | 0.187 |
| Angioplasty or stent | 116(15.4%) | 35(14.9%) | 0.861 |
| Cardiac hospitalization | 363(48.1%) | 91(38.7%) | 0.012 |
| **Etiology, n (%)** |  |  |  |
| Ischemic heart disease | 383(50.7%) | 133(56.6%) | 0.116 |
| Hypertensive | 111(14.7%) | 54(23.0%) | 0.003 |
| Dilated cardiomyopathy | 120(15.9%) | 19(8.1%) | 0.003 |
| **Medication status, n (%)** | | | |
| Beta- blockers | 478(63.3%) | 133(56.6%) | 0.064 |
| ACEIs/ARBs | 522(69.1%) | 148(63.0%) | 0.078 |
| MRAs | 447(59.2%) | 123(52.3%) | 0.063 |
| Diuretics | 487(64.5%) | 110(46.8%) | <0.001 |
| Nitrate | 257(34.0%) | 100(42.6%) | 0.018 |
| Antiplatelet drugs | 499(66.1%) | 147(62.6%) | 0.32 |
| Digoxin | 209(27.7%) | 50(21.3%) | 0.051 |
| Abbreviations: HFrEF heart failure with reduced ejection fraction, HFmrEF heart failure with mid-range ejection fraction, HFpEF heart failure with preserved ejection fraction, BMI body mass index, NYHA: New York Heart Function Assessment, MoCA Montreal cognitive assessment, SBP systolic blood pressure, DBP diastolic blood pressure, JVP jugular venous pressure, COPD chronic obstructive pulmonary disease, CKD chronic kidney disease, MI myocardial infarction, HF heart failure, CAD chronic coronary artery disease, ACEIs angiotensin-converting enzyme inhibitors, ARBs angiotensin receptor blockers, MRAs mineralocorticoid receptor antagonists | | | |
