# Supplementary material for: Clinical characteristics and prognosis of heart failure with mid-range ejection fraction: insights from a multi-centre registry study in China
Source: BMC Cardiovasc Disord. 2019 Sep 2;19:209. doi: 10.1186/s12872-019-1177-1 (PMC6720401; doi:10.1186/s12872-019-1177-1)
Supplement: Supplementary file 2 — Table S2. Predictors of 1-year events by univariate analysis. (Display of the predictors of 1-year events by univariate analysis) (DOC 67 kb) [file 12872_2019_1177_MOESM2_ESM.doc]

| **Supplementary Table 2. Predictors of 1-year events by univariate analysis** | | | | | | | | |
| --- | --- | --- | --- | --- | --- | --- | --- | --- |
|  | **All-cause Mortality** | | **Cardiovascular Mortality** | | **MACE** | | **Hospitalization Due to HF** | |
|  | **p value** | **HR (95% CI)** | **p value** | **HR (95% CI)** | **p value** | **OR (95% CI)** | **p value** | **OR (95% CI)** |
| HF Categories | **0.004** |  | **<0.001** |  | **<0.001** |  | 0.920 |  |
| HFmEF vs HFrEF | **0.032** | **0.461(0.227-0.935)** | **0.009** | **0.298(0.120-0.737)** | **0.046** | **0.492(0.245-0.987)** | 0.685 | 0.901(0.546-1.488) |
| HFmEF vs HFpEF | 0.593 | 1.233(0.572-2.660) | 0.317 | 1.782(0.574-5.533) | 0.053 | 2.303(0.991-5.353) | 0.845 | 0.956(0.605-1.509) |
| Female | 0.475 | 1.218(0.709-2.094) | 0.938 | 1.027(0.518-2.037) | 0.627 | 1.155(0.645-2.069) | 0.568 | 0.896(0.614-1.307) |
| Age | 0.564 | 1.006(0.986-1.026) | 0.814 | 0.997(0.973-1.021) | 0.693 | 0.996(0.975-1.017) | 0.592 | 1.004(0.990-1.018) |
| BMI | 0.261 | 0.960(0.895-1.031) | 0.774 | 0.988(0.907-1.075) | 0.826 | 0.992(0.923-1.066) | 0.450 | 0.982(0.938-1.029) |
| Married | 0.452 | 0.768(0.385-1.530) | 0.947 | 1.033(0.399-2.674) | 0.868 | 0.936(0.427-2.051) | 0.470 | 0.834(0.510-1.365) |
| Non-solitary | 0.121 | 0.533(0.240-1.182) | 0.110 | 0.461(0.178-1.193) | 0.087 | 0.477(0.204-1.115) | 0.804 | 1.094(0.539-2.217) |
| Medication insurance | 0.282 | 0.684(0.343-1.366) | 0.761 | 1.176(0.413-3.354) | 0.387 | 1.520(0.589-3.924) | 0.311 | 1.340(0.761-2.360) |
| Educated | 0.809 | 1.072(0.611-1.880) | 0.102 | 1.938(0.877-4.284) | 0.607 | 1.173(0.639-2.153) | 0.357 | 0.838(0.575-1.221) |
| MoCA | **0.002** | **0.943(0.908-0.979)** | 0.339 | 0.976(0.928-1.026) | 0.141 | 0.969(0.929-1.011) | **0.045** | **0.972(0.946-0.999)** |
| Pulse | 0.561 | 0.995(0.978-1.012) | 0.487 | 0.992(0.971-1.014) | 0.751 | 0.997(0.979-1.015) | **0.002** | **1.016(1.006-1.027)** |
| SBP | 0.146 | 0.990(0.976-1.004) | 0.075 | 0.984(0.967-1.002) | 0.262 | 0.992(0.978-1.006) | **0.01** | **1.011(1.003-1.020)** |
| DBP | 0.119 | 0.981(0.959-1.005) | 0.302 | 0.985(0.956-1.014) | 0.230 | 0.985(0.961-1.010) | 0.222 | 1.010(0.994-1.025) |
| NYHA(III-IV) | 0.085 | 1.693(0.930-3.079) | **0.029** | **2.534(1.102-5.827)** | **0.006** | **2.719(1.336-5.534)** | **<0.001** | **2.636(1.713-4.054)** |
| JVP(>6cmH_2_O) | **0.048** | **1.726(1.004-2.966)** | **0.015** | **2.307(1.175-4.527)** | **0.016** | **2.039(1.139-3.649)** | **<0.001** | **2.361(1.619-3.442)** |
| Hypertension | 0.775 | 0.924(0.538-1.588) | 0.595 | 0.832(0.423-1.637) | 0.708 | 0.895(0.501-1.599) | 0.596 | 1.106(0.761-1.608) |
| Diabetes Mellitus | 0.195 | 1.485(0.817-2.700) | 0.045 | 2.055(1.017-4.153) | **0.001** | **2.864(1.573-5.216)** | 0.131 | 1.395(0.906-2.149) |
| Hyperlipidemia | 0.440 | 0.786(0.427-1.448) | 0.547 | 0.791(0.369-1.696) | 0.267 | 0.684(0.350-1.337) | 0.593 | 1.113(0.751-1.649) |
| COPD | 0.765 | 0.856(0.309-2.374) | 0.953 | 1.037(0.316-3.397) | 0.708 | 1.202(0.459-3.143) | 0.499 | 1.239(0.665-2.307) |
| CKD | 0.400 | 1.486(0.591-3.739) | 0.214 | 1.942(0.682-5.526) | **0.007** | **3.085(1.360-6.999)** | **0.003** | **2.511(1.355-4.655)** |
| Stroke | 0.589 | 0.803(0.361-1.782) | 0.820 | 1.108(0.457-2.691) | **0.024** | **2.163(1.109-4.220)** | **0.030** | **1.684(1.053-2.693)** |
| Prior MI | 0.250 | 0.627(0.283-1.389) | 0.250 | 0.542(0.191-1.540) | 0.786 | 1.105(0.538-2.269) | 0.621 | 1.124(0.708-1.783) |
| Tobacco use | 0.099 | 1.574(0.918-2.699) | **0.036** | **2.067(1.050-4.072)** | **0.048** | **1.798(1.006-3.213)** | 0.928 | 1.018(0.695-1.491) |
| Family history of HF | 0.992 | 0.993(0.242-4.082) | 0.792 | 0.765(0.105-5.601) | **0.023** | **3.229(1.175-8.871)** | 0.072 | 2.102(0.936-4.724) |
| Family history of CAD | 0.573 | 0.715(0.223-2.296) | 0.689 | 0.747(0.179-3.121) | 0.547 | 1.345(0.512-3.531) | 0.077 | 1.725(0.942-3.158) |
| Cardiac hospitalization | 0.373 | 1.280(0.744-2.201) | 0.257 | 1.487(0.749-2.951) | 0.310 | 1.351(0.755-2.419) | **<0.001** | **2.420(1.644-3.563)** |
| Ischemic heart disease | 0.885 | 1.041(0.607-1.786) | 0.975 | 1.011(0.515-1.985) | **0.039** | **2.022(1.038-3.938)** | 0.220 | 0.793(0.547-1.149) |
| Dilated cardiomyopathy | 0.368 | 1.357(0.698-2.638) | 0.268 | 1.567(0.708-3.467) | 0.103 | 1.704(0.898-3.237) | 0.954 | 1.015(0.613-1.681) |
| Beta-blockers | 0.385 | 0.785(0.454-1.356) | 0.980 | 0.991(0.493-1.994) | 0.754 | 0.909(0.502-1.648) | 0.262 | 0.805(0.552-1.175) |
| ACEIs/ARBs | 0.094 | 0.627(0.362-1.083) | 0.374 | 0.730(0.365-1.462) | 0.549 | 0.830(0.451-1.527) | 0.578 | 0.894(0.602-1.328) |
| MRAs | 0.182 | 1.481(0.832-2.637) | 0.719 | 1.135(0.568-2.269) | 0.546 | 0.836(0.467-1.497) | 0.455 | 1.156(0.79-1.691) |
| Diuretics | 0.321 | 1.354(0.744-2.463) | 0.312 | 1.482(0.691-3.179) | 0.052 | 1.977(0.993-3.936) | **0.013** | **1.686(1.115-2.551)** |
| Digoxin | 0.594 | 1.174(0.651-2.115) | 0.448 | 1.323(0.642-2.729) | 0.259 | 1.422(0.772-2.620) | 0.820 | 1.049(0.695-1.582) |
| | Abbreviations: HFrEF heart failure with reduced ejection fraction, HFmrEF heart failure with mid-range ejection fraction, HFpEF heart failure with preserved ejection fraction, MACE major adverse cardiac events, BMI body mass index, NYHA: New York Heart Function Assessment, MoCA Montreal cognitive assessment, SBP systolic blood pressure, DBP diastolic blood pressure, JVP jugular venous pressure, COPD chronic obstructive pulmonary disease, CKD chronic kidney disease, AMI acute myocardial infarction, HF heart failure, CAD chronic coronary artery disease, ACEIs angiotensin-converting enzyme inhibitors, ARBs angiotensin receptor blockers, MRAs mineralocorticoid receptor antagonists.  Statistically signiﬁcant variables were highlighted in bold. | | --- | | | | | | | | | |
