# Supplementary material for: Clinical characteristics and prognosis of heart failure with mid-range ejection fraction: insights from a multi-centre registry study in China
Source: BMC Cardiovasc Disord. 2019 Sep 2;19:209. doi: 10.1186/s12872-019-1177-1 (PMC6720401; doi:10.1186/s12872-019-1177-1)
Supplement: Supplementary file 3 — Table S3. Independent predictors of 1-year events in final multivariate models by backward LR method. (Display of the independent predictors of 1-year events in final multivariate models by backward LR method) (DOC 44 kb) [file 12872_2019_1177_MOESM3_ESM.doc]

| **Supplementary Table 3. Independent predictors of 1-year events in final multivariate models by backward LR method** | | | | | | | | | | | |
| --- | --- | --- | --- | --- | --- | --- | --- | --- | --- | --- | --- |
| **All-cause Mortality** | | | **Cardiovascular Mortality** | | | **MACE** | | | **Hospitalization Due to HF** | | |
| **Variables** | **p value** | **HR (95% CI)** | **Variables** | **p value** | **HR (95% CI)** | **Variables** | **p value** | **OR (95% CI)** | **Variables** | **p value** | **OR (95% CI)** |
| HF Categories | **0.002** |  | HF Categories | **<0.001** |  | HF Categories | **0.001** |  | HF Categories | 0.283 |  |
| HFmEF vs HFrEF | **0.022** | **0.437(0.215-0.887)** | HFmEF vs HFrEF | **0.005** | **0.27(0.108-0.672)** | HFmEF vs HFrEF | **0.034** | **0.450(0.215-0.941)** | HFmEF vs HFrEF | 0.952 | 1.017(0.588-1.758) |
| HFmEF vs HFpEF | 0.488 | 1.320(0.602-2.894) | HFmEF vs HFpEF | 0.455 | 1.554(0.488-4.950) | HFmEF vs HFpEF | 0.093 | 2.138(0.882-5.183) | HFmEF vs HFpEF | 0.199 | 0.721(0.438-1.187) |
| Female | 0.067 | 2.000(0.954-4.195) | Female | **0.027** | **2.827(1.125-7.108)** | Female | **0.022** | **2.577(1.147-5.788)** | NYHA(III-IV) | **0.001** | **2.189(1.358-3.530)** |
| Non-solitary | 0.079 | 0.486(0.218-1.086) | Non-solitary | **0.050** | **0.379(0.143-1.002)** | Non-solitary | **0.037** | **0.373(0.148-0.944)** | JVP(>6cmH_2_O) | **0.002** | **1.913(1.259-2.905)** |
| MoCA | **0.006** | **0.946(0.909-0.984)** | JVP(>6cmH_2_O) | 0.060 | 1.940(0.972-3.874) | NYHA(III-IV) | **0.031** | **2.295(1.081-4.874)** | Pulse | **0.007** | **1.016(1.004-1.027)** |
| Tobacco use | **0.012** | **2.491(1.221-5.079)** | Diabetes Mellitus | 0.052 | 2.031(0.995-4.149) | Diabetes Mellitus | **0.004** | **2.607(1.366-4.976)** | SBP | **0.002** | **1.014(1.005-1.023)** |
| ACEIs/ARBs | 0.084 | 0.612(0.350-1.068) | Tobacco use | **0.007** | **3.444(1.403-8.454)** | CKD | **0.043** | **2.519(1.031-6.153)** | CKD | **0.014** | **2.326(1.182-4.577)** |
|  |  |  |  |  |  | Stroke | **0.022** | **2.346(1.129-4.872)** | stroke | 0.057 | 1.640(0.985-2.731) |
|  |  |  |  |  |  | Tobacco use | **0.005** | **3.102(1.406-6.842)** | Family history of HF | 0.065 | 1.843(0.964-3.523) |
|  |  |  |  |  |  |  |  |  | Cardiac Hospitalization | **<0.001** | **2.556(1.678-3.895)** |

Abbreviations: HF heart failure, HFrEF heart failure with reduced ejection fraction, HFmrEF heart failure with mid-range ejection fraction, HFpEF heart failure with preserved ejection fraction, MACE major adverse cardiac events, NYHA: New York Heart Function Assessment, MoCA Montreal cognitive assessment, SBP systolic blood pressure, JVP jugular venous pressure, CKD chronic kidney disease, ACEIs angiotensin-converting enzyme inhibitors, ARBs angiotensin receptor blockers.

Statistically signiﬁcant variables were highlighted in bold.
